# Lung segmentation on standard and mobile chest radiographs using oriented Gaussian derivatives filter

**DOI:** 10.1186/s12938-015-0014-8

**Published:** 2015-03-04

**Authors:** Wan Siti Halimatul Munirah Wan Ahmad, W Mimi Diyana W Zaki, Mohammad Faizal Ahmad Fauzi

**Affiliations:** Faculty of Engineering, Multimedia University, Persiaran Multimedia, Cyberjaya, Selangor Malaysia; Department of Electric, Electronic & Systems Engineering, Faculty of Engineering and Built Environment, Universiti Kebangsaan Malaysia, Bangi, Selangor Malaysia

**Keywords:** Chest radiograph, Unsupervised lung segmentation, Fuzzy C-means, Thresholding, Gaussian derivatives, Medical image processing, Segmentation algorithm

## Abstract

**Background:**

Unsupervised lung segmentation method is one of the mandatory processes in order to develop a Content Based Medical Image Retrieval System (CBMIRS) of CXR. The purpose of the study is to present a robust solution for lung segmentation of standard and mobile chest radiographs using fully automated unsupervised method.

**Methods:**

The novel method is based on oriented Gaussian derivatives filter with seven orientations, combined with Fuzzy C-Means (FCM) clustering and thresholding to refine the lung region. In addition, a new algorithm to automatically generate a threshold value for each Gaussian response is also proposed. The algorithms are applied to both PA and AP chest radiographs from both public JSRT dataset and our private datasets from collaborative hospital. Two pre-processing blocks are introduced to standardize the images from different machines. Comparisons with the previous works found in the literature on JSRT dataset shows that our method gives a reasonably good result. We also compare our algorithm with other unsupervised methods to provide fairly comparative measures on the performances for all datasets.

**Results:**

Performance measures (accuracy, F-score, precision, sensitivity and specificity) for the segmentation of lung in public JSRT dataset are above 0.90 except for the overlap measure is 0.87. The standard deviations for all measures are very low, from 0.01 to 0.06. The overlap measure for the private image database is 0.81 (images from standard machine) and 0.69 (images from two mobile machines). The algorithm is fully automated and fast, with the average execution time of 12.5 s for 512 by 512 pixels resolution.

**Conclusions:**

Our proposed method is fully automated, unsupervised, with no training or learning stage is necessary to segment the lungs taken using both a standard machine and two different mobile machines. The proposed pre-processing blocks are significantly useful to standardize the radiographs from mobile machines. The algorithm gives good performance measures, robust, and fast for the application of the CBMIRS.

## Background

Chest radiography is the most frequently used diagnostic imaging examination for chest diseases such as lung cancer, pulmonary edema (fluid in the lung), pleural effusion (fluid between lung and chest cavity), pneumonia (infection by bacteria, viruses, fungi, or parasites) and tuberculosis (bacterial infection). More than 10 million people worldwide die annually from chest diseases. Based on the survey done by [1], the mortality rates for chest diseases in 1990 are 6.3 million (ischaemic heart disease), 4.3 million (lower respiratory infections), 2.2 million (chronic obstructive pulmonary disease), 2 million (tuberculosis) and 0.9 million (lung cancer). For most diseases, many cures are only effective in the early stage and symptomless stage of disease. Screening can help early diagnosis, but a sensitive, side effect-free as well as economical method has to be used to enable mass usage. Standard chest radiography meets these requirements, except that current methods have moderate sensitivity. It is still more favourable despite the development of advances radiological exams like Computed Tomography (CT). The main reason is because the CT exams expose the patient to a higher dose of radiation. By comparing the conventional CXR and CT chest, it is estimated that the latter is about 400 times higher than the former, which equivalent to 3.6 years of background exposure [2]. Another reason for the widespread use of conventional chest radiograph over CT is its economic feasibility. The study on literature and challenges for current direction of computer aided detection (CADe) system for lung cancer in CT scans are reviewed by Firmino et al. in [3]. There are some recent works found on analysing the chest in CT images, including the pulmonary fissure detection and lobe segmentation [4-8]. However, the topic of interest for this research is only on chest radiography, thus the previous literature on related work will be discussed thoroughly in the next section.

### Previous work

Medical image segmentation plays a crucial role in many imaging applications by automating or facilitating the delineation of anatomical structures and other regions of interest. Segmentation of lung fields in CXR has received considerable attention in the literature since the past decade. An exhaustive survey on the lung segmentation techniques for chest radiographs has been done for this work, and is summarized in Table 1. Most of the listed work used JSRT dataset as their image database. JSRT dataset is the database of chest radiographs (with and without lung nodules) that publicly made available by the Japanese Society of Radiological Technology (JSRT), with their ground truth clinical data [9]. This dataset only consists of posterior-anterior (PA) chest radiographs, taken by stationary X-Ray machine. To the best of our knowledge there are only two work [10,11] that used chest radiographs from portable device. This shows the lack of study in mobile chest radiographs that is also relatively important especially for very sick patients whenever their radiographs will be taken using portable X-Ray machine.

**Table 1:** **Summary of related work on lung segmentation techniques for chest radiographs**

**Reference**	**Image database**	**Segmentation method**	**Evaluation measure**	**Limitation**
[33]	- 230 chest radiographs		Overlap score:	
		- ASM with optimal local features	- ASM right: 0.882 ± 0.074	- Computationally complex
		- find optimal displacements for landmarks using a non-linear kNN classifier instead of linear Mahalanobis distance	- ASM left: 0.861 ± 0.109	- Suffers the drawback of ASM
			- ASM-OF right: 0.929 ± 0.026	
			- ASM-OF left: 0.887 ± 0.114	
[17]	- JSRT dataset (247 images)	Various methods were compared	Overlap score:	- Highly supervised and required training
		- Hybrid voting	- Hybrid voting: 0.949 ± 0.020	
		- PC postprocessed	- PC postprocessed: 0.945 ± 0.022	
		- Hybrid ASM-PC	- Hybrid ASM-PC: 0.934 ± 0.037	
		- Hybrid AAM-PC	- Hybrid AAM-PC: 0.933 ± 0.026	
		- ASM-tuned	- ASM-tuned: 0.903 ± 0.057	
		- AAM	- AAM: 0.847 ± 0.095	
		- Mean Shape	- Mean Shape: 0.713 ± 0.075	
[10]	- 24 chest radiographs from portable device, all with pulmonary bacterial infections manifested as consolidations	- based on Bezier interpolation of salient control points	Sensitivity: 95.3%	- Lack of images
			Specificity: 94.3%	
[11]	- 58 chest radiographs from portable device, all with pulmonary bacterial infections manifested as consolidations	- Gray-level selective thresholding followed by ASM	Accuracy presented in a graph, between 92.5% - 94%.	- Lack of images
				- Suffers the drawback of ASM
[15]	- 52 selected images from JSRT dataset	- Gaussian kernel-based fuzzy clustering algorithm with spatial constraints	Accuracy:	- Lack of images (only 52 were selected out of 247 images in JSRT dataset)
			- 0.978 ± 0.0213	
[13]			Dice similarity:	- Requires training and optimization
	- 1,130 images	- rule-based method (thresholding, morphology and connected components) used to generate a seed mask	- 0.88 ± 0.07	
	- 400 from Shanghai Pulmonary Hospital (200 normal, 200 with pneumoconiosis)	- Using optimized canny edge parameters to detect the corner (costophrenic angle)		
	- 730 from different clinical sites in China (with normal and various pulmonary conditions)			
[36]	- JSRT dataset (247 images)		Overlap score:	- Requires optimization and testing
		- Fusing shape information with statistical model of the lungs’ shape	- 22 landmarks: 0.92 ± 0.063	
		- intensity-based iterative thresholding	- 28 landmarks: 0.94 ± 0.053	
		- optimization using ASM		
[34]	- JSRT dataset (247 images)	- ASM for the lung segmentation, with bone detection algorithm	- Sensitivity: 0.956	- Suffers the drawback of ASM
			- Specificity: 0.984	
[14]	- JSRT dataset (247 images)		Accuracy:	
		- based on spatial relationships between lung structures, represented as fuzzy subsets of the image space	- Left axillary: 82.1%	- Need to label the lung structures
		- segment the lung structures	- Right axillary: 85.2%	- Accuracy or overlap score of whole lung is not provided
			- Left parahilar: 84.4%	
			- Right parahilar: 82.8%	
			- Left Paracardiac: 68.8%	
			- Right Paracardiac: 86.5%	
			- Left Basal: 81.5%	
			- Right Basal: 81.7%	
[35]			Accuracy:	- Requires shape-learning stage
	- JSRT dataset (93 normal images)	- Global edge and region force (ERF) field based ASM (ERF-ASM)	- JSRT left: 0.952 ± 0.013	
	- CXR from University of Alberta Hospital dataset (50 images with tuberculosis)	- PCA analysis to learn the lung fields’ shape	- JSRT right: 0.955 ± 0.014	
			- CXR left: 0.946 ± 0.015	
			- CXR right: 0.953 ± 0.017	
[37]		3 stages:	Overlap score:	
	- JSRT dataset (247 images)	1. CBIR approach to identify small set of lung CXR using Radon transform with Bhattacharyya similarity measure	- JSRT: 0.954	- Need to be highly trained
	- Montgomery dataset (138 images – 80 normal, 58 with tuberculosis)	2. Construction of patient-specific lung atlas	- Montgomery: 0.941	- Computationally complex
	- India dataset (200 images – 100 normal, 100 abnormal)	3. Lung segmentation using graph cuts discrete optimization approach	- India: 0.917	

Column ‘Reference’ refers to the citation of previous work; column ‘Image database’ describes the image database used in the cited work; column ‘Segmentation method’ summarizes the methods used in the cited work; column ‘Evaluation measure’ listed all the performance measures available in the cited work; and column ‘Limitation’ gives the known limitation related to the cited work.

In [12], the early segmentation methods for CXR have been classified into roughly four categories: rule-based methods, pixel classification-based methods, deformable model-based methods and hybrid methods. A rule-based scheme consists of a sequence of steps, tests and rules. The methods used are thresholding (local), region growing, edge detection, ridge detection, morphological operations, fitting of geometrical models, functions or dynamic programming. The usage of rule-based scheme is demonstrated in [13], based on Bezier interpolation of salient control points is used in [10] and based on fuzzy subsets of the image space in [14]. Pixel classification-based scheme on the other hand is more general and mainly model the intensities of the image and classify the pixels into lung field or background. [15,16] employed the scheme based on fuzzy clustering method (FCM) and [17] tested the post-processed pixel classification method as one of their comparative results.

The FCM algorithm is the best known, although it has many drawbacks in applying into finding appropriate groups in data analysing problems. Many researchers have tried to modify the basic objective function to have more robust FCM [18-23]. However, the ideal segmentation of an image is usually application-dependent; and FCM has been used with some success in the soft or fuzzy segmentation in medical imaging of chest CT [7,23-28], chest MRI [18] and brain MRI [16,19-22,25,29]. In CT and MRI images, the edges of the lung or brain can be easily distinguished due to the distinct bone and cell tissue, thus motivated the authors to apply the FCM in their work. For CXR, Shi, et al. [15] has implemented FCM with spatial constrains to segment the lung, and Rastgarpour, et al. [16] has also integrated a local region-based level set method with a variation of fuzzy clustering in their work in order to segment a few modalities and body parts including lung. A few works on other applications using FCM in CXR has also been found in the literature. Parveen et al. in [30] implemented the algorithm for detection of pneumonia, [31] used for segmentation of heart information (size, contour and shape) and lastly the application is for clustering feature vectors data for atypicality detection by [32]. Lack of work on lung segmentation for CXR using FCM is mainly due to the inhomogeneities of the X-Ray imaging [16]. Furthermore, the strong edges at the rib cage and clavicle region as well as intensity variation around the lung area make it challenging to use FCM as segmentation tool.

A relatively new scheme which have been extensively studies and used in medical image segmentation is the deformable model-based methods. This shape-flexibility model, namely Active Shape Model (ASM) and Active Appearance Model (AAM), have been successfully applied to lung region segmentation [17,33-35]. However, they both have several limitations and shortcomings including requires supervision to adjust certain parameters which produces highly variable solutions, requires shape learning to train the model, as well as manual initialization. The invention of hybrid scheme is to produce better segmentation results by fusing the previous said schemes. It is very interesting to note that most of the hybrid methods found in the literature is combining rule and shape based schemes to their algorithms [11,36,37]. Still, the methods fused with ASM suffer the drawbacks and the shape scheme on the other hand needs to undergo the optimization process, learning, training and usually are computationally complex.

Computer aided evaluation of CXR needs complex image processing algorithms where the images should be pre-processed prior to the detection of abnormalities. The first step for the development of an automatic system for digital chest radiographs is the segmentation of the CXR to extract the area of the lungs before suppressing the thoracic cage (the bones). By doing this, there is a chance to eliminate shadows of these parts, cleaning the area of the lung field from the anatomical noise and making it possible to look behind the bones. Thus, this paper will focus on the lung field segmentation.

### Proposed segmentation algorithm

**Figure 1:**
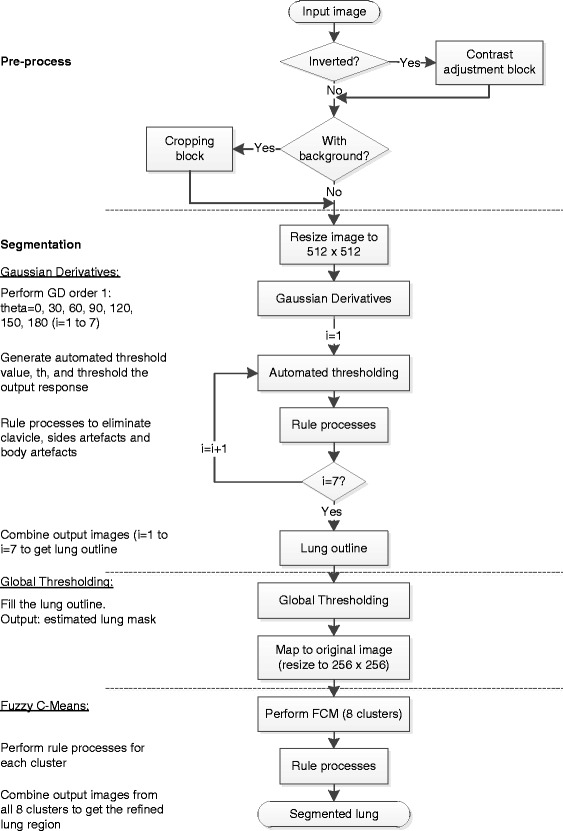
**Image processing flow for the proposed lung segmentation method.** The diagram is divided into two main sections: the pre-process (with contrast adjustment and cropping block) and segmentation (with Gaussian Derivatives, global thresholding and Fuzzy C-Means algorithms).

The proposed image segmentation method is a rule-based approach that consists of several algorithms applied sequentially. Images collected from mobile and stationary X-Ray machines produce different kind of radiographs. Thus, a robust algorithm has been proposed to overcome this issue that consists of two stages: the pre-processing stage and the segmentation stage. A schematic of the image processing flow is shown in Figure 1.

### Pre-processing

**Figure 2:**
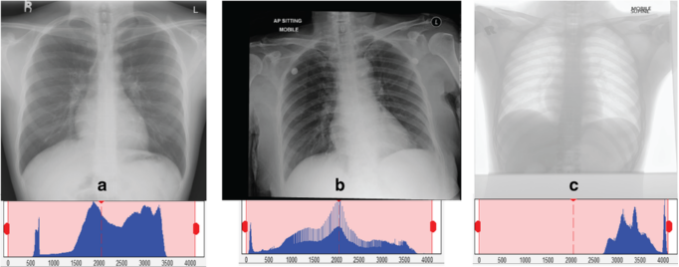
**Example of different projection and positioning in chest radiographies with their respective histograms. (a)** PA erect from standard machine; **(b)** AP sitting; and **(c)** AP Supine from portable machines.

The radiographs produced by both stationary and portable machines have diverse purposes, settings and configurations. Their varieties in position views and properties are among the main challenges to develop a robust algorithm to extract the lung. Figure 2 shows few examples of various CXR images obtained from three different machines and their respective histograms. It is obviously shown that histograms of different CXR images have different intensity distribution from each other. Figure 2(a) is an example of normal PA radiograph from a stationary machine, which is the output of common CXR machines. In this work, this type of CXR image is set as the standard image where no pre-processing step is needed.

**Figure 3:**
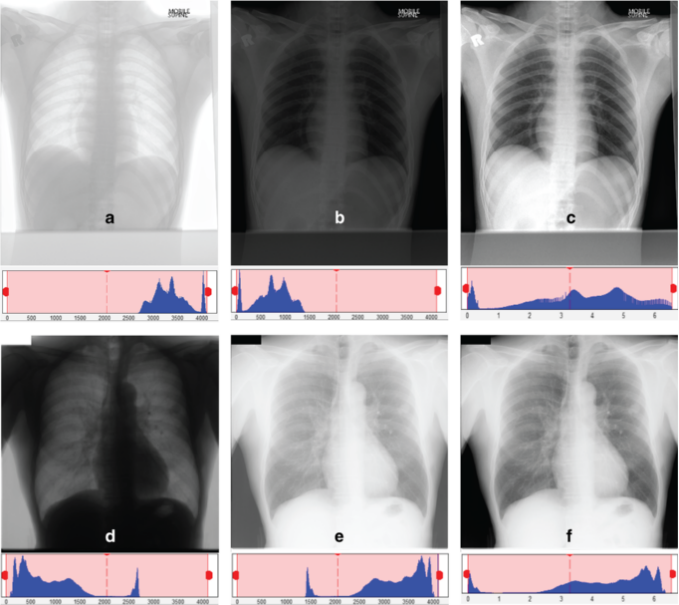
**The outputs of the contrast adjustment block.** The two images are from different portable machines **(a)** to **(c)** and **(d)** to **(f)**. **(a)** and **(d)** are the original images, **(b)** and **(e)** are after inverting the image and **(c)** and **(f)** are the results after correcting the contrast.

In addition, Figure 2(b) and Figure 2(c) are examples of CXR images taken from two different mobile machines, where the positions are usually in sitting or lying, as well as standing if a patient is able to do so. Images from the first mobile machine (i.e. Figure 2(b)) are identified ‘with unnecessary background’, thus the chest area has to be cropped out from the images. The second mobile machine generates another type of images, such as Figure 2(c) in which is considered as ‘inverted image with unnecessary background’. Therefore, its image histogram has to be inverted before cropping the chest area. The contrast of the inverted image is then corrected by stretching the histogram and eliminated 2% of the outliers. Figure 3 illustrates examples of original radiographs and their corresponding inverted images from two different datasets.

**Figure 4:**
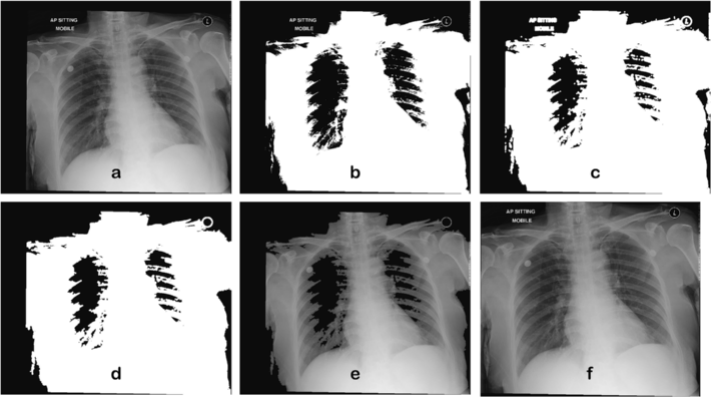
**The outputs of the cropping block. (a)** – **(f)**: original image, thresholded image, after dilation, outside wordings removal, mapped to original image, final output (cropped).

The radiographs produced by the portable machines have extra backgrounds such as in Figure 3 due to the nature of the mobile x-ray detector. Thus, cropping block is important to remove the unwanted backgrounds or regions. The radiographs are firstly converted to binary images in which their threshold value are obtained using Otsu’s thresholding method and followed by morphological dilation to preserve as much coverage as possible. Then, any wordings or unwanted regions outside the chest area are removed before cropping out the remaining background. Figure 4 shows some output images obtained from these step-by-step procedures.

Figure 1 clearly illustrates these pre-processing steps during the first stage of the proposed lung segmentation method.

### Segmentation

The proposed lung segmentation consists of a scheme that based on Gaussian derivatives (GD) filter, global thresholding and fuzzy C-means (FCM) clustering method, as clearly presented after the pre-processing stage in Figure 1. This fully automated method has adapted the oriented Gaussian filter to obtain responses in several directions so that a rough lung outline can be identified. The lung outline is then filled using global thresholding, and the final output is refined using a few different clusters of FCM. In addition, a new algorithm to get an automatic threshold value for all Gaussian filters responses has also been proposed.

#### Estimated lung outline using oriented derivatives of Gaussian filters

**Figure 5:**
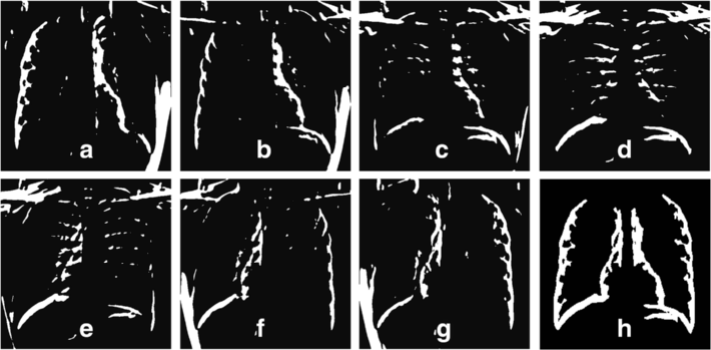
**Output of the GD responses after thresholding. (a)** – **(g)** thresholded responses for *θ=*0°,30°,60°,90°,120°,150° and 180°, and **(h)** output of the combined responses after the ‘cleaning’ processes with rule-based algorithms.

In this experimental work, an estimated outline of the lung borders is obtained by combining thresholded pixels from the output responses of oriented GD order 1 in seven directions that are *θ*=0°, 30°, 60°, 90°, 120°, 150° and 180°, at a scale of *σ*=3. Based on distributions of the gradient values, different threshold values are generated for each output responses. With a correct threshold value, the output responses of each direction may successfully depict different details of the lung outlines. For instances, at *θ*=0° and *θ*=30° , details of the outer right lung and inner left lung are highlighted, and the opposite directions (*θ*=150° and *θ*=180°) represent the details of the outer left lung and inner right lung. In addition, the response at *θ*=60° highlights the hemidiaphragm of the right lung and the inner-lower details of the left lung; while the other side of the lung can be highlighted by the response at *θ*=120°. For *θ*=90°, most of the normal PA radiographs have both right and left hemidiaphragms highlighted, whilst some of the radiographs (especially with fluid or infection) lost this feature due to the high intensity of the consolidations around the diaphragm area. Some examples of the thresholded responses are shown in Figure 5.

### Novel algorithm to get automatic threshold value for each Gaussian responses

A new method is proposed to automatically compute a threshold value for each different response by analysing its histogram obtained from the Gaussian filter.

The algorithm has been designed to obtain a threshold value that is located at the end of the histogram peaks. This is to ensure that only important gradient intensity is highlighted after the thresholding process, with as much noise reduction as possible. Equation 1 mathematically represents the histogram number of occurrences for each pixel value, called histogram numbers, *H*_*N*_. The input of this algorithm is the Gaussian response, *R*_*σ*_, with floating point pixel values where its range is varying from negative of thousands to positive thousands. The first step is to round these values to the closest integers, and the minimum and maximum values are denoted as *mn* and *mx*. The number of occurrences, *N*, for each pixel value is recorded, where each single value in the histogram is considered without discretizing it into any number of bins, and the result is called *histogram numbers, H*_*N*_.

1 $$ {H}_N(i)={\displaystyle \sum_{i=mn}^{mx}N\left({R}_{\sigma }(i)\right)} $$

The peaks of the *H*_*N*_ are calculated based on the local maxima with the minimum peak height (mph) is set to the mean of *H*_*N*_. For each peak found, *P*_*HN*_*(x)*, its location inside the vector *H*_*N*_ is denoted as *L*_*P-HN*_*(x)*, where *x*=1,2,..m; and *m* is the total number of peak found.

2 $$ {P}_{HN(x)}={H}_{N(i)}, $$

if *H*_*N*(*i* − 1)_ < *H*_*N*(*i*)_ > *H*_*N*(*i* + 1)_ and $$ {H}_{N(i)}>\frac{H_N}{mx-mn} $$

The *P*_*HN*_*(x)* are then analysed to get the chosen peak values, *P*_*HN*_*(c)*, which fulfil these criterion:

- *L*_*P-HN*_*(x)* has to be located after the maximum *H*_*N*_
- *P*_*HN*_*(x)* has to be at least 3 times of mph and at most 8 times of mph. This is to ensure that the values are to be ‘true peaks’ and above average, but not too high to exclude the ‘oddly high peaks’.

The locations of the chosen peak, *L*_*P-HN*_*(c)* are mapped to the original pixel values, and a list of possible threshold values, *L*_*TH*_, is created. Lastly, the maximum value of *L*_*TH*_ that is located at the end of the histogram peaks is chosen as the output of this algorithm.

### Rule-based algorithms

The rule-based algorithms are important steps before the lung area is segmented from its radiograph. They involve border cleaning, noise removal, clavicles elimination, and removal of body and sides’ artefacts for each thresholded response. The unwanted details that are located along the image margins are contributed by noise from the acquisition system. Some radiographs contain clavicles or body artefacts. These unnecessary details are eliminated by removing the pixels connected to the borders. The connected pixel areas which are less 0.5% of the image size are also eliminated. The three proposed rules are presented below to get the most optimum lung outlines, and clear output responses.

#### To eliminate clavicle

The clavicles are located at the top-side of the lungs; top-right for right lung and top-left for left lung, and often located close enough to the image top and sides margins. To eliminate the clavicle for the right side, a rule to filter the regions is proposed where the area with the connected pixels located between 0% and 35% of the image height, *r*_*0*_, are removed. The location of minimum row has to be at most 20% of the *r*_*0*_; and the pixels that are located between 0% and 50% of the image width, *c*_*0*_, are removed, where the location of minimum column has to be at most of 25% of the *c*_*0*_. The clavicle normally has a broad width; hence the width of the area has to be at least 15% of the image width, *c*_*0*_. Same rules are applied to the left side, except the pixels has to be located between 50% and *c*_*0*_, where the location of maximum column has to be at least 75% of *c*_*0*_.

#### To eliminate sides’ artefacts

These noises are normally caused by shadows inside the radiographs and they are located at the right or left sides of the image. The rule is carefully defined in such a way that only the most minimal side regions are removed. For the right side, the region has to be at most only 15% of *c*_*0*_, with the location of minimum column at 5% of *c*_*0*_ or lesser. Same portion of limit is applied to the left side, with the location of maximum column at least 85% of *c*_*0*_.

#### To eliminate body artefacts

This rule is proposed mainly to tackle the remaining body artefacts that are not removed during the image margin cleanings. These noises are not connected to the border, but they are located very close to the bottom image’s margins. Based on these characteristics, a rule such that, the minimum row has to be located at 25% of the *r*_*0*_ or more, and location of maximum row has to be at least 90% of the *r*_*0*_ is defined. Area of the connected pixels is set to be at least 2.5% of the image size to preserve important lung outline such as the hemidiaphragm which is normally located at the bottom of the image and very close to the margin .The limit for minimum column for the right side is set to be at most 5% of the *c*_*0*_ and the limit for maximum column for the left side is set to be at least 95% of the *c*_*0*_.

After the noise elimination steps, the output of each response is then combined to form a rough lung outline. The last rule is to eliminate small region so that only clean lung outline information is stored. The area of each connected region found in the image is calculated, and the value of largest area, *A*_*max*_, is stored. Any region that is smaller than 15% of the *A*_*max*_ and maximum column located less than 20% of *c*_*0*_ (right side) or minimum column located more than 80% of the *c*_*0*_ (left side), are discarded. The summary of this proposed rule-based algorithms are summarized in Table 2. At the end of this process, the estimated lung outline, *L*_*outline*_, as in Figure 5 (h) is obtained, and another simple algorithm based on thresholding and convex hull is developed to fill in the lung outline.

**Table 2:** **Summary of the rule-based algorithms for noise removal**

**Steps**	**Location**	**Area**
To remove small connected pixel	Anywhere	< 0.005 of image size
To eliminate clavicle	Near to top: (0 < pixel < 0.35 of *r*_*0*_) and (minimum row ≤ 0.2 of *r*_*0*_)	≥ 0.15 of *c*_*0*_
	Right lung: Top-right (0 < pixel < 0.5 of *c*_*0*_) and (minimum column ≤ 0.25 of *c*_*0*_)	
	Left lung: Top-left (0.5 of *c*_*0*_ < pixel < *c*_*0*_) and (maximum column ≥ 0.75 of *c*_*0*_)	
To eliminate sides’ artefacts	(region ≤ 0.15 of *c*_*0*_)	
	Right side: (minimum column ≤ 0.05 of *c*_*0*_)	
	Left side: (maximum column ≥ 0.85 of *c*_*0*_)	
To eliminate body artefacts	Near the bottom image’s margins: (minimum row ≥ 0.25 of *r*_*0*_) and (maximum row ≥ 0.9 of *r*_*0*_)	≥ 0.25 of image size
	Right side: (minimum column ≤ 0.05 of *c*_*0*_)	
	Left side: (maximum column ≥ 0.95 of *c*_*0*_)	
To eliminate small region after lung outline is formed	Right side: (maximum column < 0.2 of *c*_*0*_)	≤ 0.15 of *A*_*max*_
	Left side: (minimum column > 0.8 of *c*_*0*_)	

#### Fill the lung outline based on global thresholding and convex hull

**Figure 6:**
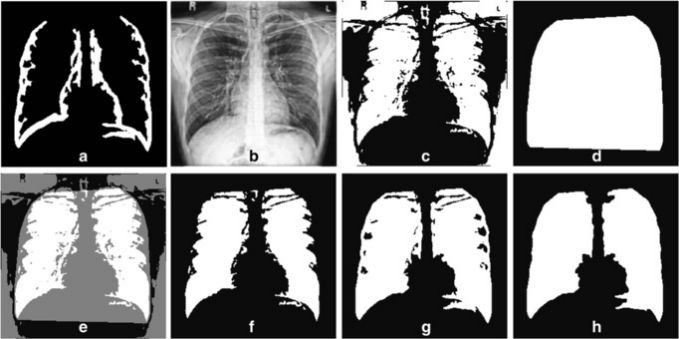
**Filling the lung outline based on global thresholding and convex hull. (a)** Input image *L*_*outline*_ **(b)** smoothed *I*_*HFEF*_, **(c)** thresholded *I*_*HFEF*_ (*I*_*th*_), **(d)** convex hull of *L*_*outline*_ (*L*_*CH*_), **(e)** ROI of *I*_*th*_ within *L*_*CH*_ **(f)** *I*_*th-roi*_, **(g)** *I*_*th-roi*_ + *L*_*outline*_, and **(h)** final estimated lung mask, *L*_*mask*_.

Figure 6 (a) and Figure 6 (b) show images obtained from previous processing steps denoted as *L*_*outline*_ and normalized image using high frequency emphasis filtering (HFEF), *I*_*HFEF*_, respectively. The *I*_*HFEF*_ is smoothed using Gaussian blurring with filter size of 32 by 32 and standard deviation, σ = 10. From our observation the chosen filter size and σ value are able to successfully smooth out the obvious edge details in *I*_*HFEF*_, and to preserve the important lung region information. A global Otsu thresholding is then applied to the smoothed *I*_*HFEF*_ and the outputs are *I*_*th*_, Figure 6 (c). A convex hull of *L*_*outline*_ is obtained and denoted as *L*_*CH*_ (Figure 6 (d)), and *I*_*th*_ is combined with *L*_*CH*_ to get the ROI of *I*_*th*_ within *L*_*CH*_ (Figure 6 (e)), hence to produce *I*_*th-roi*_ (Figure 6 (f)). The final estimated lung mask, *L*_*mask*_ (Figure 6 (g-h)), is obtained by combining *L*_*outline*_ and *I*_*th-roi*_, followed by basic morphological operations (dilation, filling, erosion and removing small regions).

#### Refine the lung region using FCM clustering

This work has proposed a rule-based algorithm using FCM clustering to further refine the lung mask. The FCM method that was improved by [38-40] is used for this purpose because of its ability to automatically cluster the pixels into the defined number of clusters. FCM has been used previously in segmenting lung in chest X-Ray [15,16], chest CT [7,23-28], chest MRI [18] as well as segmenting brain matter in MRI images [16,19-22,25,29].

**Figure 7:**
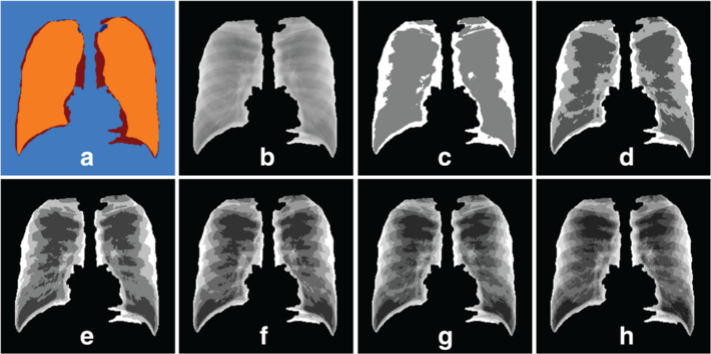
**Output of different number of clusters for FCM. (a)** highlighted ground truth region (orange) overlapped with *L*_*mask*_, **(b)** *I*_*mask*_, **(c)** *n* = 3, **(d)** *n* = 4, **(e)** *n* = 5, **(f)** *n* = 6, **(g)** *n* = 7 and **(h)** *n* = 8.

In this algorithm, input images for FCM are the estimated lung mask, *L*_*mask*_ that is illustrated in Figure 6 (h) obtained from the previous algorithm. *L*_*mask*_ is then mapped to the original CXR to produce its *I*_*mask*_ (Figure 7 (b). FCM with several numbers of clusters, *n* = 3 to 8, are tested (as shown in Figure 7) to get closest segmented lung areas as their ground truth lung regions which is overlapped with *L*_*mask*_ and highlighted as orange regions in Figure 7 (a). Figure 7 (c) to Figure 7 (h) shows the experimental results of FCM thresholding with *n* = 3 to 8 obtained for a normal patient respectively. From our observation for all cases, the output image using *n* = 8 gives the best segmentation result with the most lung information preserved.

**Figure 8:**
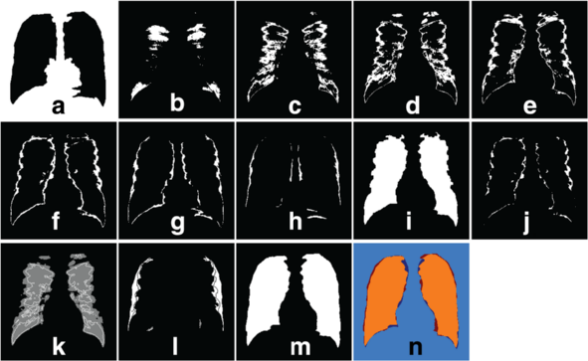
**Process of refining the lung region using FCM cluster images for n = 8. (a)** – **(h)** cluster image *I*_*c1*_, *I*_*c2*_, *I*_*c3*_, *I*_*c4*_, *I*_*c5*_, *I*_*c6*_, *I*_*c7*_, *I*_*c8*_, **(i)** processed *I*_*c5*_, **(j)** processed *I*_*c6*_, **(k)** *I*_*c234*_, **(l)** *I*_*c678*_, **(m)** final output, *L*_*final*_ and **(n)** highlighted ground truth region (orange) overlapped with *L*_*final.*_

Figure 8 (a) to Figure 8 (h) show cluster images that are to be processed denoted as *I*_*cn*_ where *n* represents the cluster from 1 to 8. From the figures, it can be seen that each cluster image has different information in which some of them can be later combined and processed together. The first cluster image, *I*_*c1*_ only contains the background information of the lung mask, thus, it will be discarded. *I*_*c2*_, *I*_*c3*_, and *I*_*c4*_ have the ‘innermost’ lung information, but in some of the imperfect output of *L*_*outline*_, these cluster images also give unwanted details such as the center bones (sternum) and connected clavicle. Nevertheless, this problem can be overcome by applying a few steps of morphological operations to the fifth cluster image, *I*_*c5*_, so that the obtained minimum lung region is then mapped to *I*_*c2*_, *I*_*c3*_, and *I*_*c4*_. Only pixels within the region of processed *I*_*c5*_ (Figure 8 (i)) are considered. The results from *I*_*c2*_, *I*_*c3*_ and *I*_*c4*_ are combined and denoted as *I*_*c234*_ (Figure 8 (k)). The next step is to process *I*_*c6*_, *I*_*c7*_, and *I*_*c8*_, where these cluster images have the outer lung details, as well as the excess of inner and lower lung noises. The connected pixels in *I*_*c6*_ is firstly eroded (Figure 8 (j)), then followed by preserving the outer lung details for *I*_*c6*_, *I*_*c7*_, and *I*_*c8*_. Any pixel falls between 40% and 60% of image width are discarded. The results of *I*_*c6*_, *I*_*c7*_ and *I*_*c8*_ are then added to form *I*_*c678*_ (Figure 8 (l)). Lastly, the refined lung region is obtained by combining *I*_*c234*_, processed *I*_*c5*_ and *I*_*c678*_ to produce the final segmented lung mask, *L*_*final*_ Figure 8 (m).

## Experiments and results

This section describes the performance evaluation of the proposed segmentation method for CXR images in our datasets. Segmentation performance has been assessed by comparing the output of proposed automated segmentation methods with the ground truth images prepared by the experts. Pixel-by-pixel analyses are performed to measure a similarity between the set of non-zero pixels of the two segmentation masks.

### Image data

For this experimental work, images from both public (JSRT) and private (SH) image datasets have been collected. Images in the former database are the standard PA chest radiographs, 247 in total and collected from 13 institutions in Japan and one in the United States. The images are a collection of normal images (93) and with exactly one nodule (154). The images were scanned from films to a size of exactly 2048 by 2048 pixels [9]. The latter database is our own private database collected from Hospital Serdang (SH), Malaysia. It contains both PA and anterior-posterior (AP) radiographs produced by three different machines: one is stationary machine to produce PA radiographs and two mobile (portable) machines specifically for ill patient which can produce both PA and AP radiographs. The database collection consists of 86 normal images and 42 images with various types of consolidations such as but not limited to fluids, infections and cavitation. The image resolutions vary and they are in DICOM file format with 12-bit grey levels.

The image projection and patient positioning in SH datasets consists of three positioning: PA erect, AP sitting and Supine. The commonly used projection in CXR are from posterior to anterior (PA), with the X-ray source situated posterior (behind) to the patient and the X-ray plate positioned immediately anterior (frontal) to the patient’s chest. If the patient is ill and unable to stand or suffers with general immobility, the CXR may be taken anterior to posterior (AP) in sitting or supine (lying) position. Only mobile machine is able to take CXR images in sitting and lying position.

### Performance measure/evaluation

The performance of the proposed segmentation algorithm is measured using a ‘goodness’ index. For two class segmentation problems such as lung and background in this work, one can distinguish true positive (TP) area (correctly classified as lung), false positive (FP) area (background incorrectly classified as lung), false negative (FN) area (lung incorrectly classified as background) and true negative (TN) area (correctly classified as background). Measures such as sensitivity, specificity, accuracy and overlap score can be computed using these values. In this work, the analyses have been performed to both right and left lungs using the following formulas:

$$ Accuracy=\frac{TP+TN}{TP+TN+FP+FN} $$

$$ OverlapScore=\frac{AreaOfInter \sec tion,TP}{AreaOfTheUnion,TP+FP+FN} $$

$$ Sensitivity/\mathrm{R}\mathrm{e} call,R=\frac{TP}{TP+FN} $$

$$ Specificity=\frac{TN}{TN+FP} $$

$$ \Pr ecision,P=\frac{TP}{TP+FP} $$

$$ F- score,F=\frac{2\times P\times R}{P+R} $$

Accuracy defines the amount of true results (both true negatives and true positives) [14]. Overlap score is defined as the agreement between the ground truth and the estimated segmentation mask over all pixels in the image [37]. This measure is well accepted and has been used widely in the lung field segmentation of chest radiographs [17]. Sensitivity or recall is corresponds to the proportion of true positives relative to the lung field that should be segmented. Sensitivity tends to 1 (or 0) if there is little (or many) false negatives [14]. Specificity is the proportion of true negatives relative to the lung field that should be segmented. Specificity tends to 1 (or 0) if there is little (or many) false positives [14]. Precision is corresponds to the proportion of true positives relative to the segmented lung field (true positives and false positives). F-Score is defined as a weighted average of both precision and recall. The best value is 1 and vice versa. Standard deviation (for all measures) is defined as the amount of disparity of the measure from its average value. The lower the standard deviation value means that the measured values are very close to their expected value. Whilst a high standard deviation value means that the measured values are varied over a large range of values. Minimum value (for all measures) defines the lowest value of the measure. A good measure shall produce a high minimum value. Maximum value (for all measures) defines the highest value of the measure. A good measure shall produce a high maximum value.

### Execution time of the proposed methods

Table 3 presents the obtained computational speed of each level in the proposed segmentation method. Different stage of level may use different image sizes, thus their respective sizes are also portrayed in the table for comparison. All algorithms are developed using Matlab 7.10 as the software tool on a desktop personal computer with a 3.10-Ghz Intel i5 CPU and 8 GB memory as a testing platform. The total execution time per dataset is slightly different; where it depends on the pre-processing steps (level number 1, 2 and 3) before the segmentation stage (refer Figure 1). Comparing the three segmentation stages (level number 4), GD stage takes the longest time, with more than 70% of the time is taken to estimate the lung outline and to generate the threshold value for each Gaussian orientation (total of 7 orientations). However, the main segmentation processes are relied on the GD method, thus, the relatively high execution time is still tolerable because the main crucial issue is to achieve high segmentation accuracy. Furthermore, the retrieval system for medical image application consists of two stages: the offline feature extraction stage and the online retrieval stage. During the offline stage, features are computed for all database images; while during the online stage, only the FV of the query image is computed. It is important to take note that the unsupervised method is mandatory for a robust retrieval system.

**Table 3:** **Average execution time for each proposed level with the respected image size**

**No**	**Level (image size)**	**Average execution time (s)**
1	Contrast adjustment block (original size)	0.68
2	Cropping block (original size)	0.15
3	Get spine axis (512 by 512 to get the spine, then reduced 256 by 256 when using HT)	0.29
4	Segmentation using GD, thresholding and FCM	13.71
	- Estimate lung outline (GD) (512 by 512)	(9.79)
	- Fill the lung outline (thresholding) (256 by 256)	(0.96)
	- Refine lung region (FCM) (256 by 256)	(2.96)

### Experiments, results and discussion

Performance of our unsupervised method will be separately evaluated for each public and private datasets in term of average (mean), standard deviation (std), minimum (min) and maximum (max) values for recall, precision and F-score performance measures. All performance measures range from 0 to 1.

#### Experiment on public image dataset (JSRT)

The JSRT dataset consists of standard PA chest radiographs from stationary machine. The radiographs are inverted images (refer Figure 3(d)), thus they have to undergo contrast adjustment process during the pre-processing stage. Table 4 presents the performance measures for this dataset. It can be seen that most performance measures are above 0.90, except the rounded overlap measure is 0.87, which is still above satisfactory. The standard deviations for all measures are also very low, which indicates the low variation of the performance measures from the mean values. The lowest value is corresponding to the specificity (0.0147) and the highest is only 0.0628 of the sensitivity. The minimum value is however quite low for certain measures, due to a few images that are difficult to segment.

**Table 4:** **Lung field segmentation for standard PA chest radiographs using the public image database (JSRT)**

	**Overlap**	**Accuracy**	**F-score**	**Precision**	**Sensitivity**	**Specificity**
mean	**0.8695**	**0.9577**	**0.9289**	**0.9332**	**0.9279**	**0.9707**
std	**0.0599**	**0.0240**	**0.0414**	**0.0327**	**0.0628**	**0.0147**
min	0.3156	0.6873	0.4798	0.7997	0.3255	0.9041
max	0.9365	0.9800	0.9672	0.9886	0.9905	0.9958

**Figure 9:**
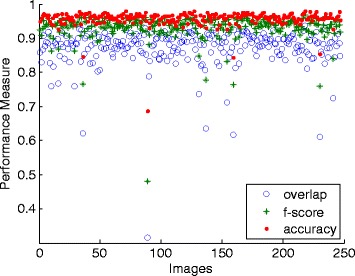
**Performance measures of the proposed method for each image using the public JSRT dataset (247 images).**

The quantitative results for this standard PA dataset are tabulated in a scattered graph as in Figure 9. The accuracy and F-score of almost all images (98% and 92% of all cases respectively) are higher than 0.90. 24% of the images achieve above 0.90 overlap score, 78% are above 0.85 and 95% are above 0.80. From the experimental work, four cases obtained about 0.60 and one case of 0.32, where the method failed to get suitable automatic threshold value when thresholding the GD responses. For these cases, the outputs led to the loss of lung outline information, thus affecting the rest of the segmentation process. This problem is due to the difference in the responses intensity, where their fit threshold values are located slightly to the centre of the histogram peaks.

**Figure 10:**
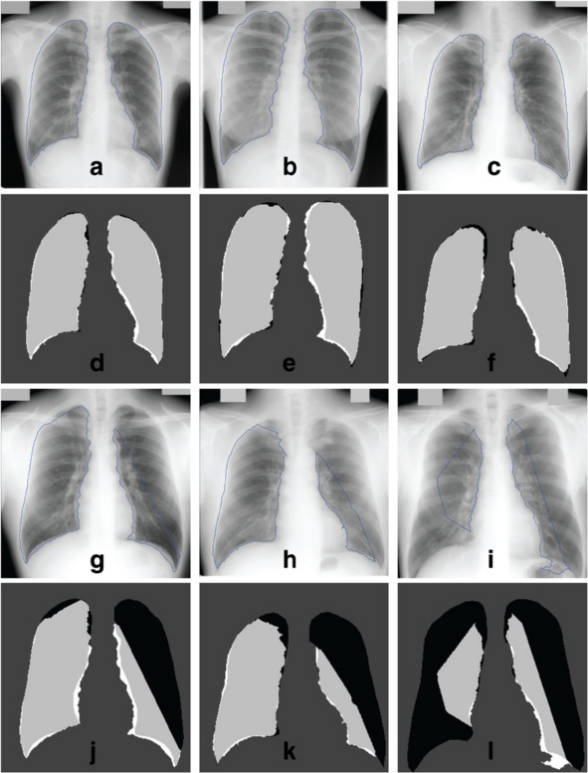
**Segmentation outputs (contours and confusion matrix) using the public JSRT dataset.** Results are shown for the best (**(a)** to **(f)**) and worst (**(g)** to **(l)**) 3 of 247 images. TN pixels are dark grey, TP are light grey, FP are white and FN are black.

Figure 10 shows the qualitative segmentation results that are the lung contours generated by the proposed method superimposed on the original images ((a) to (c) and (g) to (i)), together with the confusion matrix ((d) to (f) and (j) to (l)), corresponding to the best and worst three segmentation outputs.

#### Performance comparison on JSRT dataset with existing methods

Several studies have reported their lung segmentation results on various chest radiographs as comprehensively presented in the literature. In this work, we had compared the performance measures between the proposed method and previous studies as summarized in Table 5. We only chose the quantitative results that were achieved using all 247 images of JSRT dataset. By comparing the overlap score with the supervised hybrid-based methods in [17,36,37], our method performs reasonably well with the overlap difference of 0.07 to 0.084 and 6 to 8.5 times faster execution time. In addition, our proposed method is an unsupervised and fully automated where no training or learning stage is necessary. The proposed method also performs favourably with the supervised model-based method in [34] with the sensitivity and specificity difference of 0.028 and 0.013, respectively. Comparing with the rule-based method presented in [14], where the segmentation was done based on the lung structures and extensive knowledge was needed to label each structure, our method performs much better with the accuracy difference of 0.142. The proposed approach is unsupervised, low complexity and computationally tolerable, yet provides satisfactory results.

**Table 5:** **Segmentation methods for comparison (for 247 images in JSRT database)**

**Reference**	**Method**	**Complexity**	**Overlap**	**Sensitivity**	**Specificity**	**Accuracy**	**Execution Time**
Proposed method	Rule	Unsupervised and fully automated	0.870 ± 0.059	0.928	0.971	0.958	10-15 s (512 by 512)
[14]	Rule	Labelling	N/A	N/A	N/A	0.816	N/A
[34]	Model	Supervised	N/A	0.956	0.984	N/A	N/A
[17]	Hybrid: Model + pixel	Supervised	0.949 ± 0.020	N/A	N/A	N/A	N/A
[36]	Hybrid: Model + rule	Supervised	0.94 ± 0.053	N/A	N/A	N/A	N/A
[37]	Hybrid: Rule + Shape	Supervised	0.954 ± 0.015	N/A	N/A	N/A	85-90s (512 by 512)

#### Experiment on private datasets (SH)

To test the robustness of our unsupervised segmentation method, we have tested the algorithm using a private chest radiographs database collected from Hospital Serdang, Malaysia. Images in the private SH database contains both PA and AP chest radiographs, obtained from three different machines: one standard stationary machine (Siemens FD-X) and the other two are the mobile machines (ADC5146 and CR0975). The images from Siemens FD-X machine are the standard radiograph and do not need any pre-processing prior to the segmentation. Whilst the images from the two mobile machines have very different properties: CR0975 are with unnecessary background, thus need to be pre-processed by the cropping block; and ADC5146 are inverted image with unnecessary background, and need to be pre-processed by both contrast adjustment and cropping block.

The performance measures for standard PA chest radiographs from the private dataset (Siemens FD-X) are shown in Table 6. The segmentation results using the JSRT dataset outperforms the private dataset by 0.06 of the overlap measure. This is due to the homogenous intensity appearance of JSRT dataset, especially after pre-processed by the contrast adjustment block, whilst Siemens FD-X dataset performs above average despites not being pre-processed.

**Table 6:** **Lung field segmentation for standard PA chest radiographs using the private image database (SH: Siemens FD-X)**

	**Overlap**	**Accuracy**	**F-score**	**Precision**	**Sensitivity**	**Specificity**
mean	**0.8084**	**0.9381**	**0.8922**	**0.8607**	**0.9340**	**0.9395**
std	**0.0723**	**0.0297**	**0.0468**	**0.0626**	**0.0765**	**0.0379**
min	0.5931	0.8540	0.7446	0.6594	0.6491	0.7890
max	0.9071	0.9750	0.9513	0.9493	0.9986	0.9868

**Figure 11:**
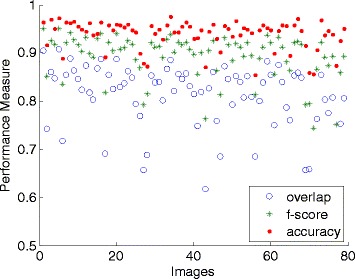
**Performance measures of the proposed method for each image using the private SH: Siemens FD-X dataset (79 images).**

**Figure 12:**
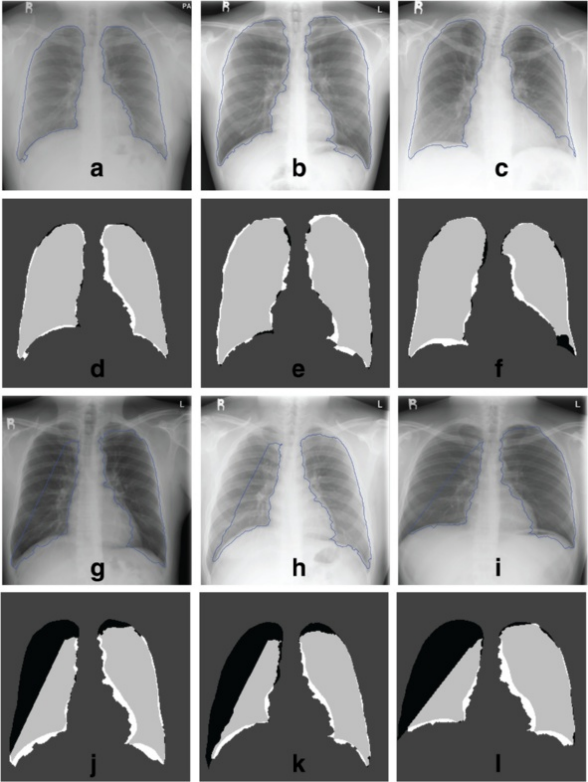
**Segmentation outputs (contours and confusion matrix) using the private Siemens FD-X dataset.** Results are shown for the best (**(a)** to **(f)**) and worst (**(g)** to **(l)**) 3 of 79 images. TN pixels are dark grey, TP are light grey, FP are white and FN are black.

The quantitative results for the private PA datasets are shown in Figure 11. Performance measures for this dataset can be loosely compared with those obtained from the JSRT dataset. Strict comparison is not possible since they were applied on different sets of data. It can be seen that the accuracy for 85% of the images are higher than 0.90, and the F-score gives 58% of measure above 0.90 and 92% are above 0.80. The overlap score achieves above 0.80 for 71% of the whole image, thirteen images having scores between 0.70 and 0.79, nine images with 0.60 to 0.69 and one with the lowest score of 0.59. The same cause as JSRT dataset can be deduced as the culprit of the low overlap measures. Besides, the overall performance of the private dataset is affected by the heterogeneous intensity of the images, while the public dataset contains images of good technical quality. The segmentation results are visually presented in Figure 12, with the lung contours generated by the proposed method superimposed on the original images, together with the confusion matrix, corresponding to the best and worst three segmentation outputs.

Table 7 presents the performance measures for mobile images obtained from two portable machines which contain both PA and AP radiographs from the private image database (CR0975 and ADC5146). The overall performances of both datasets are below average, due to most abnormalities present in the radiograph. The former dataset contains 14 abnormal cases (out of 34 images), and 9 from 12 images in the latter dataset are abnormal. However, the segmentation outputs show that almost all images from both datasets have their bottom lung border (at least one side of the lung) around the costophrenic angle are detected. A normal chest has almost symmetry shape (except the heart area in the left lung) with same level of costophrenic angle, thus, this information can be used to detect the consolidations.

**Table 7:** **Lung field segmentation for mobile PA and AP chest radiographs using the private image database (SH: CR0975 and ADC5146)**

	**Overlap**	**Accuracy**	**F-score**	**Precision**	**Sensitivity**	**Specificity**
**SH: CR0975**						
mean	**0.6902**	**0.9020**	**0.7958**	**0.8149**	**0.8393**	**0.9258**
std	**0.2049**	**0.0642**	**0.1795**	**0.0996**	**0.2532**	**0.0604**
min	0.1227	0.7287	0.2186	0.5527	0.1310	0.7458
max	0.8827	0.9677	0.9377	0.9910	0.9985	0.9989
**SH: ADC5146**						
mean	**0.6977**	**0.9195**	**0.8121**	**0.8555**	**0.8095**	**0.9501**
std	**0.1472**	**0.0442**	**0.1252**	**0.0841**	**0.2004**	**0.0532**
min	0.2841	0.8183	0.4424	0.6773	0.3021	0.8047
max	0.8328	0.9698	0.9088	0.9543	0.9978	0.9928

**Figure 13:**
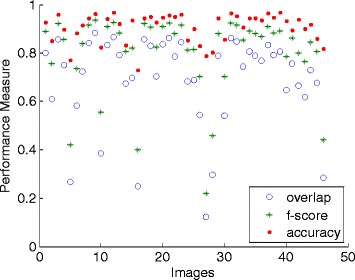
**Performance measures for each image of both private mobile datasets (CR0975 and ADC5146) with 46 images in total.**

**Figure 14:**
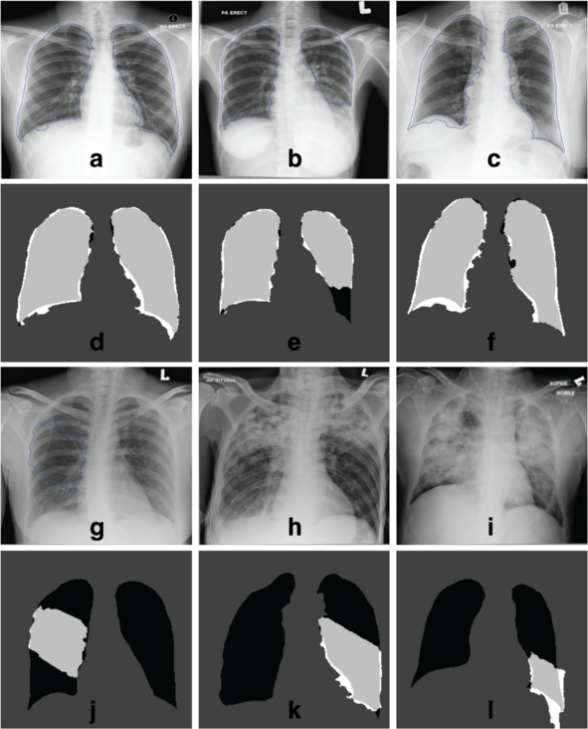
**Segmentation outputs (contours and confusion matrix) on combined private mobile dataset (CR0975 and ADC5146).** Results are shown for the best (**(a)** to **(f)**) and worst (**(g)** to **(l)**) 3 of 46 images. TN pixels are dark grey, TP are light grey, FP are white and FN are black.

Figure 13 illustrates the quantitative results of the segmentation performance using the private database. The three best and worst segmentation outputs are qualitatively presented in Figure 14. The lung contours are superimposed on the pre-processed image instead of original image. From the results, we can see that 16 images achieve overlap score above 0.80, 28 images are above 0.70, and the worst 6 are below 0.50, generated from AP radiographs. It is interesting to note that the top 13 scores are mostly from mobile PA radiographs, and the last 15 scores are all AP images. This shows that the proposed method is more suitable to segment out the lung field in PA radiographs, either acquired by mobile or standard X-Ray machine.

#### Performance comparison on all datasets with other unsupervised methods

In this section, we compare the proposed unsupervised method with other commonly used unsupervised segmentation methods: the Fuzzy C- Means (FCM) clustering and Otsu thresholding. Clustering using FCM is widely used for lung segmentation in CT thorax because of the distinct bone and cell tissue. For lung segmentation in CXR, we use four clusters FCM with combinations of several morphological operations. This work has applied Otsu’s thresholding with slope information based on the pixels distributions. In addition, it is also combined with few sequential morphological operations to clear the image from the remaining noises and refine the lung edges. The results are compared with the proposed method using both datasets (public and private) to see the effectiveness of all unsupervised methods. The results are presented in Table 8.

**Table 8:** **Lung field segmentation using other unsupervised methods (FCM and thresholding)**

	**Overlap**	**Accuracy**	**F-score**	**Precision**	**Sensitivity**	**Specificity**
**FCM on JSRT**						
mean	**0.7896**	**0.9334**	**0.8783**	**0.9202**	**0.8514**	**0.9652**
std	**0.1031**	**0.0277**	**0.0734**	**0.0475**	**0.1175**	**0.0257**
min	0.2513	0.8239	0.4017	0.6417	0.2589	0.7669
max	0.9048	0.9723	0.9500	0.9897	0.9882	0.9982
**FCM on SH: Siemens FD-X**						
mean	**0.8011**	**0.9396**	**0.8865**	**0.8796**	**0.9044**	**0.9530**
std	**0.0904**	**0.0250**	**0.0626**	**0.0813**	**0.0869**	**0.0309**
min	0.4459	0.8312	0.6168	0.5071	0.4672	0.8224
max	0.9082	0.9804	0.9519	0.9707	0.9822	0.9896
**FCM on SH: CR0975**						
mean	**0.7282**	**0.9146**	**0.8353**	**0.8243**	**0.8661**	**0.9316**
std	**0.1282**	**0.0448**	**0.1041**	**0.0871**	**0.1540**	**0.0413**
min	0.2725	0.7511	0.4283	0.6013	0.3326	0.8295
max	0.8724	0.9618	0.9318	0.9739	0.9879	0.9932
**FCM on SH: ADC5146**						
mean	**0.4941**	**0.8601**	**0.6464**	**0.6062**	**0.7245**	**0.8895**
std	**0.1581**	**0.0627**	**0.1547**	**0.1721**	**0.1823**	**0.0675**
min	0.2045	0.7227	0.3396	0.3398	0.3021	0.7340
max	0.6571	0.9250	0.7931	0.8571	0.9108	0.9733
**Thresholding on JSRT**						
mean	**0.7293**	**0.8963**	**0.8396**	**0.7876**	**0.9117**	**0.8875**
std	**0.0980**	**0.0443**	**0.0686**	**0.0944**	**0.0853**	**0.0648**
min	0.3865	0.7381	0.5575	0.4352	0.4738	0.6374
max	0.9206	0.9682	0.9587	0.9526	0.9973	0.9815
**Thresholding on SH: Siemens FD-X**						
mean	**0.7232**	**0.8993**	**0.8361**	**0.7514**	**0.9539**	**0.8789**
std	**0.0908**	**0.0364**	**0.0624**	**0.0993**	**0.0422**	**0.0522**
min	0.4931	0.8017	0.6605	0.4981	0.7510	0.7499
max	0.8953	0.9665	0.9448	0.9304	0.9967	0.9741
**Thresholding on SH: CR0975**						
mean	**0.6376**	**0.8479**	**0.7667**	**0.6738**	**0.9294**	**0.8167**
std	**0.1578**	**0.0862**	**0.1290**	**0.1505**	**0.1278**	**0.1200**
min	0.2182	0.6509	0.3582	0.3444	0.2526	0.5813
max	0.8810	0.9687	0.9367	0.9306	0.9999	0.9770
**Thresholding on SH: ADC5146**						
mean	**0.4502**	**0.8209**	**0.6075**	**0.5233**	**0.7777**	**0.8271**
std	**0.1423**	**0.0676**	**0.1492**	**0.1711**	**0.1949**	**0.0796**
min	0.1346	0.6709	0.2373	0.1884	0.3206	0.7339
max	0.6230	0.9150	0.7677	0.7306	0.9548	0.9647

From the overall results of both FCM and thresholding, we can see that FCM performs better for all datasets. The differences of the overlap measures between these two methods are 0.060 for JSRT, 0.078 for Siemens FD-X, 0.091 for CR0975 and 0.044 for ADC5146 datasets. The accuracy and specificity of all datasets are above 0.90 when using FCM, except the second mobile CXR machine (ADC5146), which is considerably high at above 0.86. Other performance measures are more than 0.80 for all datasets except the same ADC5146. This is due to the poor image quality in the dataset and most of the images contain severe diseases that cause lung shape deformation. The results from thresholding method give similar pattern, where poorer results are obtained for ADC5146 dataset.

By comparing the overlap measures for FCM and the proposed method, most of the datasets perform better with the proposed method, except on mobile machine CR0975 where FCM is higher by 0.038. For other datasets, our method is higher by 0.08 for JSRT, 0.007 for Siemens FD-X, and significantly higher on ADC5146 which is by 0.204. The accuracy and specificity of the method are above 0.90 for all datasets, and other performance measures are above 0.80, including the rounded F-score measure for CR0975 dataset. The proposed method also recorded low standard deviation measure, and high minimum and maximum measures on most datasets. This portrays that our unsupervised algorithm is more robust and perform reasonably better with any CXR dataset, either the standard PA or mobile PA and AP radiographs.

## Conclusions

This paper has realized a novel lung segmentation algorithm for chest radiographs including the image pre-processing stages with contrast adjustment and cropping blocks to standardize the images especially for the radiograph acquired by the mobile machines. The main contribution of this paper lies in the use of fully automated proposed segmentation method to isolate the lung field from PA and mobile AP chest radiographs for the application of CBMIRS. The technique is based on Gaussian oriented derivatives filter with integration of FCM and thresholding to refine the lung outline. Another novel algorithm to generate an automatic threshold value for each orientation responses was also proposed. Our proposed method is fully automated, unsupervised and no training or learning stage is necessary.

We also compared the proposed method with the existing methods from the literature on the public JSRT dataset, and applied other unsupervised methods based on FCM and Otsu thresholding to compare the results with both public and private datasets. Our method gives better performance measures on standard PA radiographs, with overlap score and accuracy of 0.870 and 0.958 respectively for JSRT dataset, and 0.808 and 0.938 for Siemens FD-X dataset. The measures from JSRT compares satisfactorily with the existing methods from the literature. For mobile PA and AP radiographs, both datasets performs below average with any of the unsupervised methods, due to the most abnormalities present. For CR0975 dataset, highest overlap score and accuracy are obtained using FCM based approach, where the measures are 0.728 and 0.915 respectively. Whilst for ADC5146 dataset, the performance measures are significantly higher using our proposed method; 0.698 and 0.920 for both overlap and accuracy. Even though the algorithm fails to accurately segment the lung field in mobile radiographs, information on general lung outline can still be used to detect the consolidations in the lung field, which will be studied in our next work. In addition, the ultimate goal of this work is to incorporate the proposed method in the Content-based Medical Image Retrieval System (CBMIRS) for Chest X-Ray. It is important to take note that the unsupervised method is mandatory for a robust retrieval system.
